# FODMAP-Targeting Digestive Enzyme Blend for Management of Gastrointestinal Symptoms: A “Real-World” Pre-Post Intervention Cohort Study

**DOI:** 10.1016/j.gastha.2026.100898

**Published:** 2026-02-13

**Authors:** Alexander J. Kaye, Sarah R. Meyers, David Hachuel, Jocelyn Wells, Thomas Wallach, Savanna Thor

**Affiliations:** 1 Division of Gastroenterology and Hepatology, SUNY Downstate Medical Center, Brooklyn, New York; 2 Department of Psychiatry, Rutgers Robert Wood Johnson Medical School, Piscataway, New Jersey; 3 Kiwi Biosciences, Cambridge, Massachusetts; 4 Division of Pediatric Gastroenterology, SUNY Downstate Health Sciences University, Brooklyn, New York

**Keywords:** Irritable Bowel Syndrome, Gut–Brain Interaction, Abdominal Pain, FODMAPs

## Abstract

**Background and Aims:**

Irritable bowel syndrome (IBS) is a highly prevalent and morbid condition that causes tremendous symptom burden, impacts quality of life, and generates substantial healthcare costs. Current therapies are challenging to utilize and do not provide relief to many patients, creating a clear need for new innovation. In this study, we assess the efficacy of fermentable oligosaccharides, disaccharides, monosaccharides, and polyols (FODMAP)-hydrolyzing digestive enzymes in controlling IBS symptoms.

**Methods:**

We present a single-arm, open-label pre-post intervention cohort study among patients who purchased FODMAP-targeting enzymes online. Participants completed anonymous online surveys capturing demographics and symptom severity at baseline and 4-week follow-up.

**Results:**

After 4 weeks using the FODMAP-targeting digestive enzyme blend, 78.0% (95% confidence interval [CI]: [69.7%, 84.5%]) reported improvements in bloating and flatulence, 75.0% (95% CI: [61.2%, 85.1%]) reported improvements in diarrhea (IBS-D only), 72.7% (95% CI: [51.9%, 86.9%]) reported improvements in constipation (IBS-C only), and 65.3% (95% CI: [56.3%, 73.2%]) reported improvements in abdominal pain. Significant improvements in overall IBS symptom severity were observed (*P* < .01). For specific IBS symptoms, the greatest improvements were seen in bloating and flatulence (*P* < .01) and abdominal pain (*P* < .01). Quality of life (*P* < .01), mental well-being (*P* < .01), and food avoidance behaviors (*P* < .01) all significantly improved as well.

**Conclusion:**

While further research is needed, this is a promising first report that enzymatic digestion may have the potential to decrease the burden of disorders of the gut–brain interaction, including IBS.

## Graphical abstract

**Figure:**
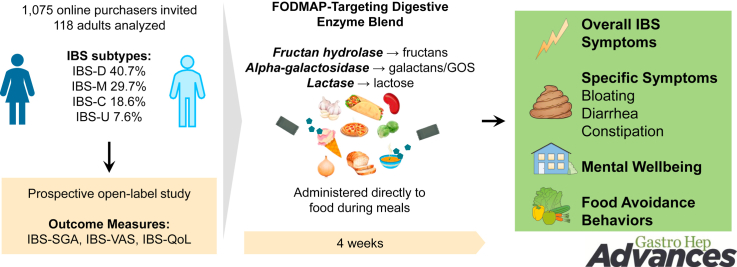

## Introduction

Disorders of the gut–brain interaction (DGBI) are a group of disorders that are defined by persistent or recurrent gastrointestinal symptoms.^1^ While there are different diagnoses and diagnostic criteria for DGBI, the Rome IV criteria are widely accepted as the primary definitions and means of diagnosing DGBI.^2^ As of 2022, functional dyspepsia and irritable bowel syndrome (IBS) are the 2 most common types of DGBI,^3^ with IBS prevalence continuing to rise in the aftermath of the COVID-19 pandemic.^4^

In addition to high (and increasing) prevalence, IBS creates a notable burden on quality of life and healthcare costs. In the United States, the annual IBS-specific spending per IBS patient per year during 2016–2021 ranged from $922 to $2,222.^5^

While IBS is not linked with significant mortality, the associated medical and psychiatric morbidity is highly prevalent. Not only is there a relative paucity of effective therapeutic interventions, but providers also face challenges in matching treatments to patients. Significantly higher levels of anxiety and depression in patients with IBS also influence selection and response to treatment.^6^

One of the most effective approaches studied is a diet low in fermentable oligosaccharides, disaccharides, monosaccharides, and polyols (FODMAP).^7^ However, of the FODMAPs, not all are equally likely to drive symptoms. While FODMAP triggers vary by patient, prior work suggests that fructans are the FODMAP component most associated with IBS symptoms.^8^, ^9^, ^10^ The most common type of fructan fibers found in food is inulin-like fructans.^11^^,^^12^ Humans do not contain the necessary enzymes to hydrolyze fructans into fructose and only an estimated 5%–15% are absorbed in the small intestine.^13^ The fructans that pass into the colon are fermented by the bacteria within the colon and contribute to IBS symptoms.^12^^,^^13^ FODMAPs are theorized to increase the luminal water content and increase fermentation by enteric bacteria, with secondary distention of the lumen and aberrant interpretation of signal driving pain and symptoms.^13^ While a low FODMAP diet has been shown to lower global IBS symptoms, its restrictive nature can be challenging and increase risk for nutritional deficiencies, eating disorders, and other negative psychosocial consequences.^7^ To avoid excessive restriction and prevent possible micronutrient deficiencies associated with a low FODMAP diet, guidelines recommend diet liberalization to the maximum extent possible, even among those who find the diet effective for symptom control.^7^

Multiple over-the-counter enzymatic products, such as lactase and alpha-galactosidase, which respectively breakdown lactose and oligosaccharides, have been on the market for those who are symptomatic when these carbohydrates are ingested. These products demonstrate the utility of assisted digestion in relieving secondary symptoms.^14^^,^^15^ In fact, guidelines on how to implement the low FODMAP diet into gastroenterological and nutrition practice recommend the use of digestive enzymes for symptom management and to allow for more dietary flexibility during diet personalization.^16^^,^^17^ However, while most enzymatic products target single FODMAP carbohydrates, the average IBS patient is sensitive to 2.5 (±2) FODMAP groups.^10^ Furthermore, fructans are known to be the most common FODMAP triggers and are highly prevalent in a typical diet, but an evidence-based enzyme solution for fructans has been elusive until now.

The FODMAP-targeting digestive enzyme blend studied is a proprietary FODMAP-targeting digestive enzyme blend of 3 active enzymes, fructan hydrolase, lactase, and alpha-galactosidase, which has shown in vitro efficacy at reducing gas and acid production in a simulated human intestine.^18^ The FODMAP-targeting digestive enzyme blend is administered directly to food to increase homogenization between FODMAPs and the enzymes, therefore maximizing FODMAP hydrolysis. This predigestion of FODMAP content before progression to the colon is intended to avoid subsequent FODMAP fermentation by the colonic bacteria.^11^ While the previous study established the efficacy of the FODMAP-targeting digestive enzyme blend in an in vitro environment, there have been no studies exploring the impact of use in a human population.^19^

We report the results herein of a pre-post intervention cohort study evaluating the impact of the FODMAP-targeting digestive enzyme blend on the mental and physical well-being of patients with IBS.

## Materials and Methods

The study was a prospective, single-arm, open-label interventional cohort study evaluating symptom changes before and after FODMAP-targeting enzyme use, with participants serving as their own controls through paired pre-post measurement. This approach was selected to capture “real-world” outcomes. The total duration of the study was 9 months, from January 23, 2024, to September 27, 2024.

### Exposure Definition

Participants who were at least 18 years old and who used the FODMAP-targeting digestive enzyme blend at least once per week for 4 weeks were eligible for inclusion. Study participants were passively recruited after purchasing FODMAP-targeting enzymes online. Participants completed an anonymous online survey capturing demographics and baseline symptom severity. Baseline was defined as the period following product purchase and prior to product use.

### Outcome Definition

The online survey consisted of the IBS-Subject’s Global Assessment (IBS-SGA), visual analog scale for IBS (VAS-IBS), and the Food Avoidance subscale of IBS quality of life (IBS-QoL).^20^, ^21^, ^22^ Clinical significance was defined as a change of 30% or more from baseline based on industry guidelines.^23^ The questions derived from these questionnaires and their associated scoring systems are displayed in Supplement 1. Demographic data included age, sex, body mass index, IBS status, and IBS subtype. Participants who reported a formal IBS diagnosis and those who highly suspected they had IBS were included, based on the question “Have you ever been diagnosed with Irritable Bowel Syndrome (IBS)?” Those who responded “Yes, I received a formal diagnosis from a clinician” were categorized as those with a formal diagnosis, and those who responded “No, but I strongly suspect I have it” were categorized as those who highly suspected they had IBS.

The IBS-SGA and VAS-IBS questionnaires are widely used to investigate symptoms in the IBS population. Both the IBS-SGA and VAS-IBS, which have been validated to assess symptom severity and psychological well-being in the IBS population, have demonstrated strong sensitivity to interventions for IBS.^20^^,^^21^ The IBS-SGA and VAS-IBS capture both overall symptom severity and specific symptoms. The Food Avoidance subscale of the IBS-QoL was also used to specifically assess changes in vigilance and frustration around food, which are primary concerns with restrictive, therapeutic diets.^22^ The IBS-QoL notably has other subscales; however, to avoid repetition in the type of data collected from study participants, only the Food Avoidance subscale was selected, as it represents an important outcome not already captured by the other IBS surveys being utilized. Prior literature has supported the ability to interpret independent IBS-QoL subscales.^24^

Four weeks after completing the initial survey, participants were invited to complete a follow-up survey that included the same IBS-SGA, VAS-IBS, and IBS-QoL measures collected at baseline. VAS-IBS scales were collected using a 0–100 scoring system, with a score of 0 indicating the highest levels of symptomatology, while 100 would indicate a complete lack of symptoms. For the IBS-QoL and IBS-SGA, symptom scoring was obtained in units from 1 to 5 (1 indicating the least frequent symptoms, and 5 indicating the most frequent symptoms) as per questionnaire instructions. Participants who used the FODMAP-targeting digestive enzyme blend less than once per week and for fewer than 4 weeks were excluded.

### Statistical Methods

All analyses assessed within-person change from baseline to the 4-week follow-up, with each participant serving as their own control. Continuous variables are presented as means with standard deviations, and categorical variables as frequencies with percentages.

For continuous outcomes, including VAS-IBS measures (abdominal pain; diarrhea among IBS-D; constipation among IBS-C; bloating/flatulence; vomiting/nausea; mental wellbeing; and gastrointestinal symptoms’ impact on daily life), IBS-SGA scores, and the IBS-QoL food avoidance composite score, baseline and follow-up values were compared using paired *t*-tests. For each outcome, the mean difference and 95% confidence interval (CI) were calculated.

For binary responder outcomes defined as achieving ≥30% improvement from baseline, proportions were calculated with 95% Wilson CIs, and changes in paired proportions were evaluated using McNemar’s test.

A Bonferroni correction was applied across the 14 prespecified primary comparisons, and statistical significance was interpreted using this adjusted threshold. All hypothesis tests were 2-sided. All analyses were conducted in Python 3.9, using pandas for data management, scipy.stats for paired tests, and statsmodels for CIs and McNemar’s test.

## Results

**Figure 1:**
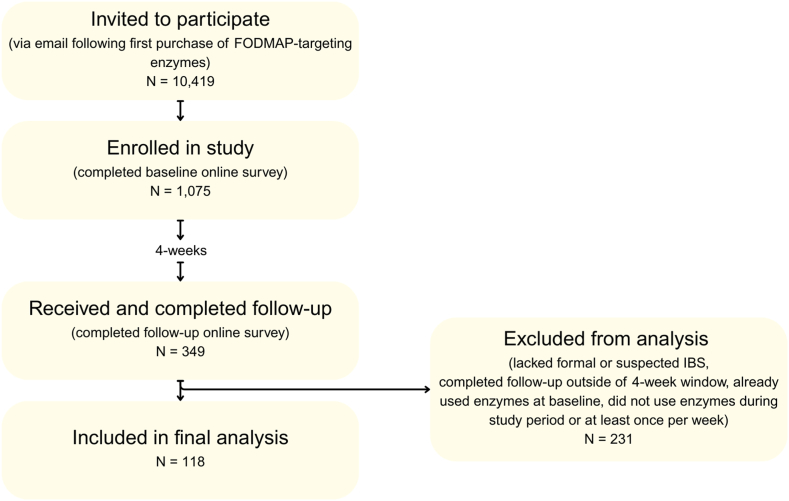
Participant flow and sample derivation. Flow of participants from initial email invitation following purchase of FODMAP-targeting enzymes through enrollment, follow-up completion, and analysis. Of the 10,419 invited customers, 1075 enrolled, 349 completed follow-up, and after applying exclusion criteria, 118 were included in the final analysis.

A total of 1075 participants took the baseline survey, and 349 completed the follow-up survey. Participants were then excluded for the following reasons: 60 participants lacked a formal or suspected IBS diagnosis; 111 did not complete the survey at the 4-week follow-up; 20 reported existing use of the FODMAP-targeting digestive enzyme blend at baseline; 5 did not use the enzyme blend during the study period; and 35 did not use the enzyme blend at least once per week for 4 weeks (Figure 1). The remaining 118 participants were included in the analysis (mean age 58 ± 14.5 years), and most participants (86.4%) were female. The mean body mass index of participants was 25.0 (±4.6). The majority had been formally diagnosed with IBS (74.6%), and the remainder highly suspected they had IBS. When evaluated for IBS subtype, 40.7% reported diarrhea-predominant IBS (IBS-D), 29.7% reported mixed IBS (IBS-M), 18.6% reported constipation-predominant IBS (IBS-C), 7.6% reported undefined IBS (IBS-U), and 3.4% reported their IBS subtype was unknown (Table 1).

**Table 1:** Study Demographics

Demographic	Category	Overall
Patient who completed the study, number		118 patients
Age, mean (SD)		58.0 years old (14.5 years old)
Sex, number (%)	Female	102 patients (86.4%)
	Male	16 patients (13.6%)
Body mass index, mean (SD)		25.0 (4.6)
Irritable bowel syndrome diagnosis, number (%)	Suspected	30 patients (25.4%)
	Yes	88 patients (74.6%)
Irritable bowel syndrome subtype, number (%)	Constipation	22 patients (18.6%)
	Diarrhea	48 patients (40.7%)
	Mixed	35 patients (29.7%)
	Undefined	9 patients (7.6%)
	Unknown	4 patients (3.4%)

SD, standard deviation.

### Evaluation of Gastrointestinal Symptoms

Table 2 shows the percent of participants reporting improvement in digestive symptoms after using the FODMAP-targeting digestive enzyme blend at least once per week. At follow-up, 72% (95% CI: [63.3%, 79.3%]) of participants reported a significant improvement in overall digestive symptoms, as measured by the IBS-SGA. Specific digestive symptoms, as measured by the VAS-IBS, also improved at clinically significant levels. Among participants, 78.0% (95% CI: [69.7%, 84.5%]) reported improvements in bloating and flatulence, 75.0% (95% CI: [61.2%, 85.1%]) reported improvements in diarrhea (IBS-D only), 72.7% (95% CI: [51.9%, 86.9%]) reported improvements in constipation (IBS-C only), and 65.3% (95% CI: [56.3%, 73.2%]) reported improvements in abdominal pain.

**Table 2:** Percentage of Patients Improving With FODMAP-Targeting Digestive Enzyme Blend

Outcomes	All (N = 118)	IBS-C (N = 22)	IBS-D (N = 48)	IBS-M (N = 35)
	% [95% CI]	% [95% CI]	% [95% CI]	% [95% CI]
More than a 30% change in VAS-IBS bloating/flatulence	78.0 [69.7, 84.5]	86.4 [66.7, 95.3]	70.8 [56.8, 81.8]	80.0 [64.1, 90.0]
More than a 30% change in VAS-IBS diarrhea	—	4.5 [0.8, 21.8]	75.0 [61.2, 85.1]	48.6 [33.0, 64.4]
More than a 30% change in VAS-IBS constipation	—	72.7 [51.9, 86.9]	27.1 [16.6, 41.0]	57.1 [40.9, 72.0]
More than a 30% change in VAS-IBS abdominal pain	65.3 [56.3, 73.2]	72.7 [51.9, 86.9]	58.3 [44.3, 71.2]	74.3 [57.9, 85.8]
More than a 30% change in VAS-IBS vomiting and nausea	28.0 [20.7, 36.7]	31.8 [16.4, 52.7]	29.2 [18.2, 43.2]	25.7 [14.2, 42.1]
Improved VAS-IBS mental wellness	65.3 [56.3, 73.2]	63.6 [43.0, 80.3]	72.9 [59.0, 83.4]	62.9 [46.3, 76.8]
Improved VAS-IBS gastrointestinal symptoms impact on daily life disruptions	73.7 [65.1, 80.8]	77.3 [56.6, 89.9]	75.0 [61.2, 85.1]	77.1 [61.0, 87.9]
Improved IBS-SGA overall FODMAP-targeting digestive enzyme blend effect	72.0 [63.3, 79.3]	63.6 [43.0, 80.3]	77.1 [63.5, 86.7]	65.7 [49.2, 79.2]
Improved IBS quality of life score	70.3 [61.6, 77.8]	59.1 [38.7, 76.7]	72.9 [59.0, 83.4]	71.4 [55.0, 83.7]

Reported as % of participants in each group meeting each outcome.

**Figure 2:**
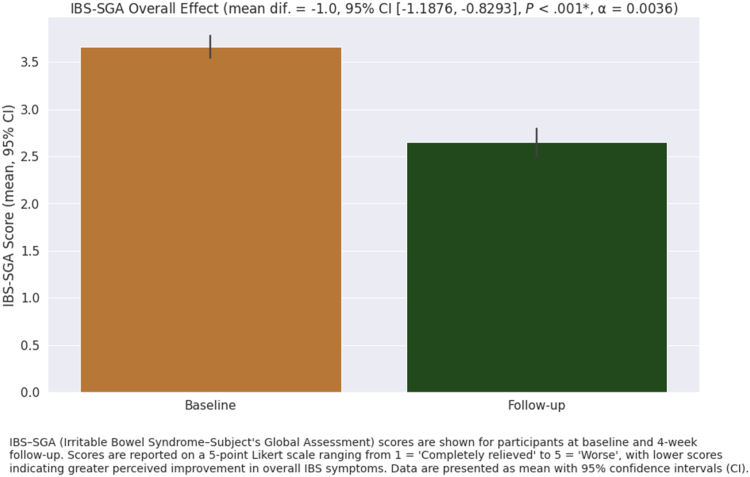
IBS-SGA scores at baseline and 4-week follow-up. IBS-SGA (irritable bowel syndrome–Subject’s Global Assessment) scores are shown for participants at baseline and 4-week follow-up. Scores are reported on a 5-point Likert scale ranging from 1 = “completely relieved” to 5 = “worse,” with lower scores indicating greater perceived improvement in overall IBS symptoms. Data are presented as mean ± standard deviation (SD).

**Figure 3:**
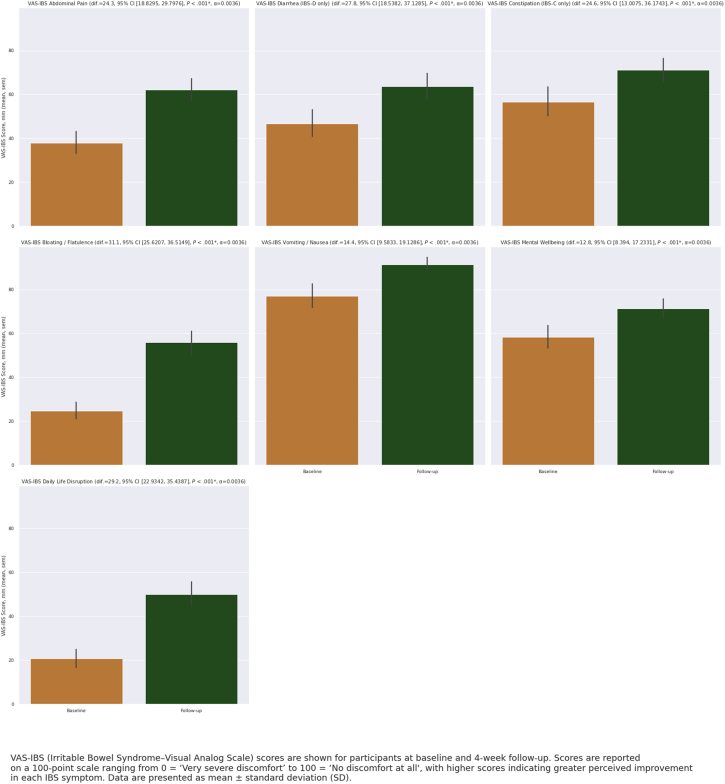
VAS-IBS scores at baseline and 4-week follow-up. VAS-IBS (visual analog scale–irritable bowel syndrome) scores are shown for participants at baseline and 4-week follow-up. Scores are reported on a 100-point scale ranging from 0 = “very severe discomfort” to 100 = “no discomfort at all,” with higher scores indicating greater perceived improvement in each IBS symptom. Data are presented as mean ± standard deviation (SD).

Significant improvements in symptom severity were observed as measured by the IBS-SGA score between baseline and follow-up (*P* < .01) (Table 3, Figure 2). For specific symptoms, the greatest improvements were seen in bloating and flatulence (*P* < .01, 95% CI: [25.6, 36.5]), for which the mean score increased by 31.1 points on the VAS-IBS scale (Figure 3). The mean VAS-IBS abdominal pain score also increased by 24.3 (*P* < .01, 95% CI: [18.8, 29.8]) (Figure 3).

**Table 3:** Outcomes Pre- and Post-Treatment With FODMAP-Targeting Digestive Enzyme Blend

Measure	Paired sample *t*-test				
	Baseline average	Follow-up average	Mean difference [95% CI]	Test statistic	*P* value^a^
VAS-IBS abdominal pain	37.8	62.1	24.3 [18.8, 29.8]	−8.78	<.001
VAS-IBS diarrhea^b^	27.1	54.9	27.8 [18.5, 37.1]	−6.02	<.001
VAS-IBS constipation^b^	18.7	43.3	24.6 [13.0, 36.2]	−4.41	<.001
VAS-IBS bloating/flatulence	24.7	55.8	31.1 [25.6, 36.5]	−11.30	<.001
VAS-IBS vomiting and nausea	77	91.4	14.4 [9.6, 19.1]	−5.96	<.001
VAS-IBS mental wellness	58.4	71.2	12.8 [8.4, 17.2]	−5.74	<.001
VAS-IBS gastrointestinal symptoms impact on daily life	20.7	49.9	29.2 [22.9, 35.4]	−9.25	<.001
IBS-SGA overall effect of FODMAP-targeting digestive enzyme blend	3.7	2.7	−1.0 [−1.2, −0.8]	11.15	<.001
IBS-QoL composite score	77.8	57.5	−20.3 [−25.2, −15.4]	8.19	<.001
IBS-QoL monitoring food amounts	3.7	3.1	−0.6 [−0.8, −0.4]	4.98	<.001
IBS-QoL monitoring food type	4.6	3.7	−0.9 [−1.1, −0.7]	8.18	<.001
IBS-QoL frustration with food	4.1	3.1	−1.0 [−1.2, −0.7]	6.84	<.001

N = 118 unless otherwise noted.

a Bonferroni correction applied to the *P* value.

b VAS-IBS diarrhea assessed only among the 48 patients with IBS-D and VAS-IBS constipation assessed only among the 22 patients with IBS-C.

### Subgroup Analysis of Gastrointestinal Symptoms

When evaluated by subtype, significant improvements in symptoms were also seen among IBS-C and IBS-D. Among the 22 participants with IBS-C, VAS-IBS constipation scores increased by 24.6 (*P* < .01, 95% CI: [13.0, 36.2]). Among the 48 participants with IBS-D, VAS-IBS diarrhea scores increased by 27.8 (*P* < .01, 95% CI: [18.5, 37.1]).

Among the 16 participants with a formal IBS-C diagnosis, VAS-IBS constipation scores increased by 27.6 (*P* = .001, 95% CI: [12.6, 42.5]). Among the 37 participants with a formal IBS-D diagnosis, VAS-IBS diarrhea scores increased by 26.1 (*P* < .01, 95% CI: [15.3, 36.9]). Among all 88 participants with a formal IBS diagnosis, VAS-IBS abdominal pain scores increased by 23.1 (*P* < .01, 95% CI: [16.5, 29.7]) and VAS-IBS bloating/flatulence scores increased by 28.5 (*P* < .01, 95% CI: [22.1, 35.0]).

VAS-IBS constipation and VAS-IBS diarrhea scores did not improve significantly among the 6 participants who suspected they had IBS-C or 11 participants who suspected they had IBS-D, though this was likely due to the small sample size (*P* = .097; *P* = .006, respectively). However, among the 30 total participants who suspected they had IBS, VAS-IBS abdominal pain scores increased by 27.8 (*P* < .01, 95% CI: [17.7, 37.9]) and VAS-IBS bloating/flatulence scores increased by 38.5 (*P* < .01, 95% CI: [28.5, 48.4]).

### Quality of Life and Mental Well-Being Measures

**Figure 4:**
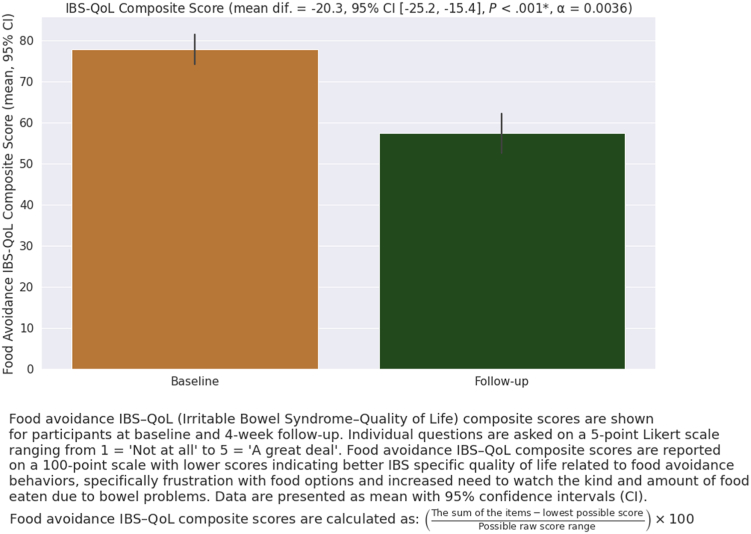
IBS-QoL scores at baseline and 4-week follow-up. Food avoidance IBS-QoL (IBS–quality of life) composite scores are shown for participants at baseline and 4-week follow-up. Individual questions are asked on a 5-point Likert scale ranging from 1 = “not at all” to 5 = “a great deal.” Food avoidance IBS-QoL composite scores are reported on a 100-point scale with lower scores indicating better IBS specific quality of life related to food avoidance behaviors, specifically frustration with food options and increased need to watch the kind and amount of food eaten due to bowel problems. Food avoidance IBS-QoL composite scores are calculated as: ([the sum of the items − lowest possible score]/possible raw score range) × 100. Data are presented as mean ± standard deviation (SD).

Quality of life and mental well-being also improved with use of the FODMAP-targeting digestive enzyme blend. At follow-up, 73.7% (95% CI: [65.1%, 80.08%]) reported improvement in the level to which their gastrointestinal (GI) symptoms disrupted their daily life and 65.3% (95% CI: [56.3%, 73.2%]) reported improvement in their mental well-being. Furthermore, quality of life improved among the majority of participants, as measured by the IBS-QoL food avoidance subscore (70.3%, 95% CI: [61.6%, 77.8%]) (Table 2, Figure 4).

Significant improvements were also seen in the level to which their GI symptoms disrupted daily life (*P* < .01) and mental well-being (*P* < .01) between baseline and follow-up (Table 3). Quality of life scores decreased (indicating improvement) by a mean of −20.3 (*P* < .01, 95% CI: [−25.2, −15.4]), representing a significant reduction in food avoidance behaviors (Figure 3). Specifically, this represents a significant improvement in the level to which their bowel problems lead them to watch the amount (*P* < .01) and kind of food they eat (*P* < .01), as well as a reduction in feelings of frustration around what they cannot eat (*P* < .01) (Table 3).

### Adverse Events

None of the study participants reported any adverse outcomes.

## Discussion

IBS is a highly (and increasingly) prevalent disease with substantial associated medical and psychiatric morbidity and costs. While some treatments exist, the highly dynamic and variable nature of the condition, along with multiple likely contributing pathways driving symptoms, collectively creates a gap in therapeutics. One of the major historical challenges in this disease space has been a relatively unclear pathophysiology driving IBS. However, in recent years, our understanding of the molecular underpinnings of DGBI pathology has increased substantially. One highly agreed upon mechanism in IBS is the impact of distention or “stretch” as a source of pain and symptoms; therefore, interventions targeting this specific problem, such as the low FODMAP diet, likely impact disease processes at a higher level by reducing triggers. It stands to reason that interventions providing a similar effect (decreased gas and acid production leading to decreased distention) would create similar results.

In this study, we present the first human data regarding the use of a fructan-digesting agent (fructan hydrolase) as a therapeutic effort to reduce the burden of IBS. The findings from this study support our hypothesis that enzymatic predigestion would improve symptoms, with all outcomes demonstrating symptomatic improvement, and 78% of patients reporting more than a 30% improvement in bloating (the symptom most directly physiologically linked to FODMAP reduction).^9^ This response rate is comparable to the average response rate to the low FODMAP diet restriction phase, which ranges from 50% to 80% among patients with IBS.^20^ In the setting of prior mechanistic work demonstrating that fructan hydrolase effectively predigests fructans under human gastrointestinal conditions, reducing acid and gas production while not eliminating butyrate production, this finding supports the therapeutic efficacy of enzymatic predigestion of FODMAPs as a therapeutic intervention in IBS and likely other DGBI responsive to a low-FODMAP diet. We additionally demonstrate improvement in symptom relief in all domains except nausea/vomiting, which is potentially explained by a more central or gastric source of these symptoms, rather than a distention-mediated process. We also are able to report perceived secondary benefits of FODMAP digestive enzymes in the form of improvement to quality of life and mental wellness.

A major challenge in treating IBS and DGBI more generally is the interaction between side effect severity and risk and the severity of the disease. Furthermore, prescription medications lead to clinical benefits in fewer than one-half of patients and provide therapeutic gains over placebo of just 7%–14%.^25^ FODMAP-targeting enzymes are generally regarded as safe substances, determined by the Food and Drug Administration as safe for human consumption. One concern with the use of enzymatic digestion could be a paucity of available short-chain fatty acids (like butyrate) for colonocyte metabolic use. However, our prior work assessing this FODMAP-targeting enzyme formulation in a simulated human intestine demonstrated relative sparing of high-value short-chain fatty acids, mitigating long-term concern for use.^18^ No participants reported adverse events during the study; the most notable negative experience described was incomplete relief of IBS symptoms when consuming FODMAP-containing foods.

This study had multiple notable limitations. First, as a single-arm, open-label, prospective pre-post intervention study, patients actively sought out this treatment, so there is likely a strong bias toward perceived efficacy. Second, we are reliant on patient reporting as well as characterization of their own diagnosis. Third, the fact that not all patients may fit Rome criteria assessment of IBS, and the inclusion of self-reported diagnosis constrains our findings somewhat. Fourth, the limited time horizon and lack of a control constrain our ability to discuss long-term efficacy and safety, and the design itself is only able to report on relative risk shifts, meaning our findings may not be generalizable. On a related note, given the absence of a control arm, the study is susceptible to regression to the mean as the study participants may have enrolled during acute symptom flares that would have improved, regardless of treatment use. Fifth, the study is the 4-week period of time between receiving the introductory survey and the trial conclusion survey, which may introduce a component of recall and reporting biases of IBS symptoms and any possible adverse effects from the FODMAP-targeting digestive enzymes. Sixth, the demographics are tilted more toward older women and IBS-D than population-level rates. Seventh, the limited survey response rate from baseline to follow-up (11%) may impact the generalizability of the data, introducing the possibility for a selection bias. For example, participants who did not perceive benefit or experience a potential adverse effect from treatment may be less inclined to complete a follow-up survey. Other measures known to influence IBS symptoms, such as mood symptoms, stress, diet, or seasonal changes, were not controlled for, nor was it feasible to obtain any objective measures. Finally, the analysis could not adjust for any possible concurrent behavioral or clinical treatments the study participants were involved in, limiting the confidence that the observed results are solely due to the studied enzyme supplement. Despite these limitations in mind, our strong positive results across symptom domains suggest potential value for this therapeutic approach, although additional controlled and long-term studies are needed to further evaluate the efficacy of these enzymatic supplements.

## Conclusion

These findings support enzymatic predigestion of FODMAPs as a possible promising alternative to strict dietary restrictions, allowing patients to enjoy a broader range of foods with fewer symptoms. The FODMAP-targeting digestive enzyme blend studied may alleviate the severity of IBS symptoms—including bloating, diarrhea, constipation, and abdominal pain—while supporting mental well-being and quality of life. While limited by the nature of the study, our findings provide encouraging real-world evidence for the role of FODMAP-targeting digestive enzymes as a tool for IBS management and demonstrate the need for a prospective randomized controlled trial of this approach.

## Supplementary Materials

Supplementary Table 1

Extended PDF
