# Supplementary material for: FODMAP-Targeting Digestive Enzyme Blend for Management of Gastrointestinal Symptoms: A “Real-World” Pre-Post Intervention Cohort Study
Source: Gastro Hep Adv. 2026 Feb 13;5(4):100898. doi: 10.1016/j.gastha.2026.100898 (PMC13018943; doi:10.1016/j.gastha.2026.100898)
Supplement: Supplementary Table 1 [file mmc1.pdf]

Supplemental Table – QOL Score

Supplement

Question number	*Question*	*Units of scoring*
1.	How disturbing during the last 30 days were your problems concerning your abdominal pain? (VAS-IBS abdominal pain)	Symptom scoring in unit from 0 to 100 (0 indicating severely symptomatic and 100 indicating no symptoms)
2.	How disturbing during the last 30 days were your problems concerning your diarrhea? (VAS-IBS diarrhea)	Symptom scoring in unit from 0 to 100 (0 indicating severely symptomatic and 100 indicating no symptoms)
3.	How disturbing during the last 30 days were your problems concerning your constipation? (VAS-IBS constipation)	Symptom scoring in units from 0 to 100 (0 indicating severely symptomatic and 100 indicating no symptoms)
4.	How disturbing during the last 30 days were your problems concerning your bloating and flatulence? (VAS-IBS bloating/flatulence)	Symptom scoring in units from 0 to 100 (0 indicating severely symptomatic and 100 indicating no symptoms)
5.	How disturbing during the last 30 days were your problems concerning your vomiting and nausea? (VAS-IBS vomiting and nausea)	Symptom scoring in units from 0 to 100 (0 indicating severely symptomatic and 100 indicating no symptoms)
6.	How do you rate your mental well-being over the past 30 days? (VAS-IBS mental wellness)	Symptom scoring in units from 0 to 100 (0 indicating severely symptomatic and 100 indicating no symptoms)

7.	How much/little have your gastrointestinal problems influenced your daily life over the past 30 days? (VAS-IBS gastrointestinal_symptoms_impact on daily life disruptions)	Symptom scoring in unit from 0 to 100 (0 indicating severely symptomatic and 100 indicating no symptoms)
8.	Have you, during the last 30 days, felt urgency to defecate? (VAS-IBS urgency to defecate)	Score of 0 indicated the lack of symptoms, and score of 1 indicated the presence of symptoms
9.	Have you, during the last 30 days, felt that your bowel has not been completely empty after visiting the toilet? (VAS-IBS incomplete evacuation)	Score of 0 indicated the lack of symptoms, and score of 1 indicated the presence of symptoms
10.	Please think about your life over the past 30 days, and look at the statements below: I have to watch the amount of food I eat because of my bowel problems? (IBS- QoL monitoring food amounts)	Symptom scoring in units from 1 to 5 (1 indicating least frequent symptoms, and 5 indicating the most frequent symptoms)
11.	Please think about your life over the past 30 days, and look at the statements below: I have to watch the kind of food I eat because of my bowel problems? (IBS- QoL monitoring food type)	Symptom scoring in units from 1 to 5 (1 indicating least frequent symptoms, and 5 indicating the most frequent symptoms)
12.	Please think about your life over the past 30 days, and look at the statements below: I feel frustrated that I cannot eat when I want because of my bowel problems? (IBS- QoL frustration with food)	Symptom scoring in units from 1 to 5 (1 indicating least frequent symptoms, and 5 indicating the most frequent symptoms)
13.	Please consider how you felt this past week in regard to your overall well-being, and symptoms of abdominal discomfort, pain, and altered bowel habits. How would you rate your relief of symptoms during the past week? (IBS-SGA overall FODZYME® effect)	Symptom scoring in units from 1 to 5 (1 indicating least frequent symptoms, and 5 indicating the most frequent symptoms)
